# Neuroendocrine tumor of the ampulla of Vater treated with endoscopic papillectomy: A case report

**DOI:** 10.1002/deo2.191

**Published:** 2022-11-27

**Authors:** Koichi Hamada, Atsushi Irisawa, Yoshinori Horikawa, Yoshiki Shiwa, Kae Techigawara, Masafumi Ishikawa, Takayuki Nagahashi, Daizo Fukushima, Noriyuki Nishino, Hideo Sakuma, Michitaka Honda

**Affiliations:** ^1^ Department of Gastroenterology Southern‐Tohoku General Hospital Fukushima Japan; ^2^ Department of Minimally Invasive Surgical and Medical Oncology Fukushima Medical University Fukushima Japan; ^3^ Department of Gastroenterology Dokkyo Medical University School of Medicine Tochigi Japan; ^4^ Department of Pathology Southern‐Tohoku General Hospital Fukushima Japan; ^5^ Department of Surgery Southern‐Tohoku General Hospital Fukushima Japan

**Keywords:** ampulla of vater, duodenoscopes, endoscopy, neoplasms, neuroendocrine tumors

## Abstract

We report the case of a 62‐year‐old female with a 6.3‐mm low‐grade neuroendocrine tumor of the ampulla of Vater, who underwent an endoscopic papillectomy. An endoscopic papillectomy was performed without complications. In the 26 months of follow‐up, no local recurrence or metastasis occurred. Endoscopic treatment of ampullary neuroendocrine tumors is controversial. However, endoscopic papillectomy may be considered a treatment option if neuroendocrine tumors are small (<10 mm), have a low grade (G1), or do not have muscle layer or bile duct invasion.

## INTRODUCTION

Neuroendocrine tumors (NET) of the ampulla of Vater are sporadic and comprise <0.3% of gastrointestinal NETs.^1^ The frequency of duodenal NETs involving the Vater papillae is increasing owing to improved instrument quality.^2^ Surgical resection with lymphadenectomy (Whipple's resection) is indicated for treating NETs in the Vater region but is invasive.^3^ Endoscopic papillectomy is an accepted therapy for intramucosal ampullary neoplasms. However, endoscopic papillectomy has not been established as a standard treatment for NETs in Vater's area because of the high risk of lymph node metastasis and the minimal number of cases. NETs in the Vater region have a worse prognosis than non‐papillary duodenal NETs, whereas low‐grade NETs have a better prognosis.^3^, ^4^ Therefore, there are several reports of endoscopic resection for small lesions of 10 mm and low‐grade ampullary NETs. However, evidence is lacking, and there is still controversy regarding treatment options.^5^, ^6^

We present a case of a small, low‐grade ampullary NET that was successfully treated with endoscopic papillectomy with no recurrence in 26 months.

## CASE REPORT

**FIGURE 1:**
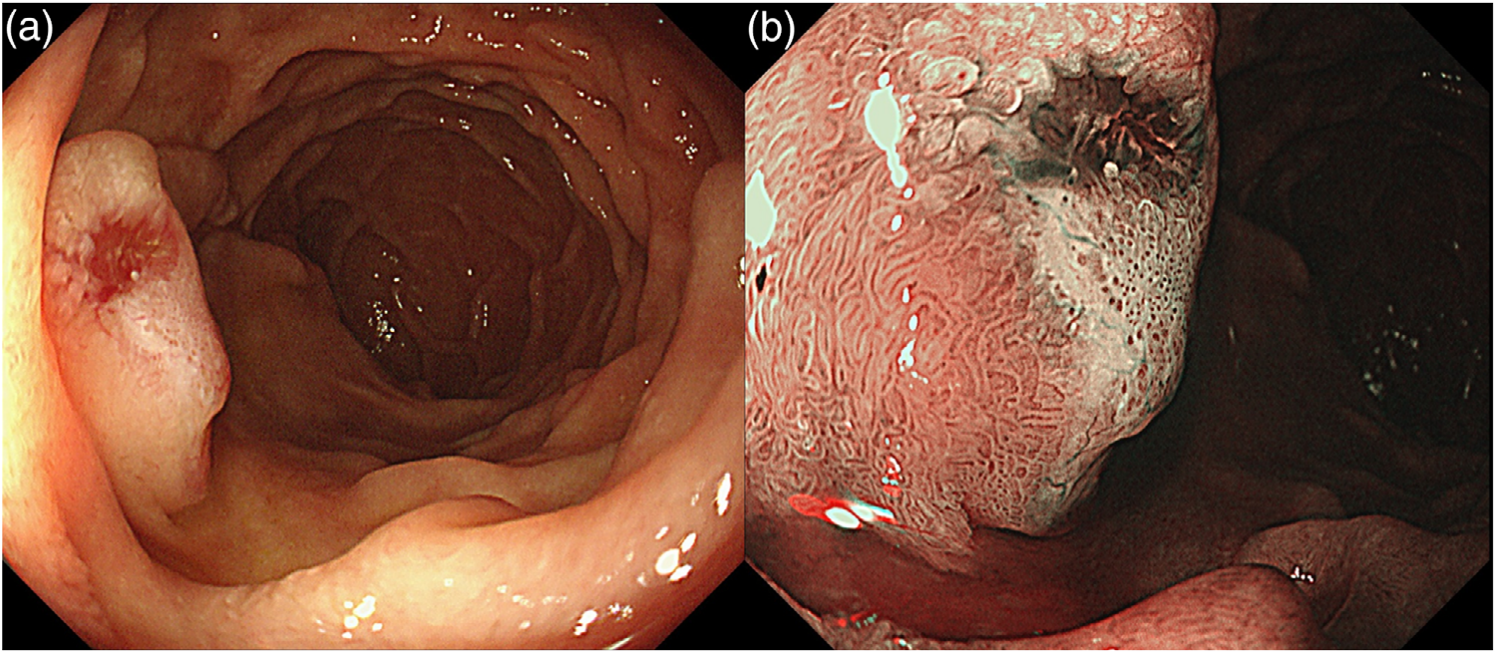
Endoscopic images of the ampulla of Vater. (a) A white light image showing a 6‐mm tumor resembling a submucosal tumor. (b) The narrow band image shows prominent vascular dilatation

A 62‐year‐old woman who underwent esophagogastroduodenoscopy as part of a physical examination was found to have an enlarged ampulla of Vater. The patient was suspected of having a NET on histological examination and was admitted to our hospital. No hormone‐related symptoms were observed. She had a medical history of diabetes mellitus and asthma and surgical history of cesarean section. Laboratory testing revealed no abnormalities (serum aspartate aminotransferase, 21 U/L; alanine aminotransferase, 26 U/L; alkaline phosphatase, 225 U/L; total bilirubin, 0.5 mg/dl; amylase, 104 U/L; white blood cell count, 6940/μl; red blood cell count, 411 × 10^4^/μl; and platelet count, 28.7 × 10^4^/μl). Scrutiny esophagogastroduodenoscopy performed at our hospital revealed enlargement of the ampulla of Vater. Narrow‐band imaging magnification revealed well‐defined small round pits and prominent vascular dilatation (Figure 1). A biopsy was performed, and histopathological analysis confirmed a low‐grade NET. Endoscopic ultrasound, contrast‐enhanced multidetector computed tomography, and positron emission tomography were performed for further examination.

**FIGURE 2:**
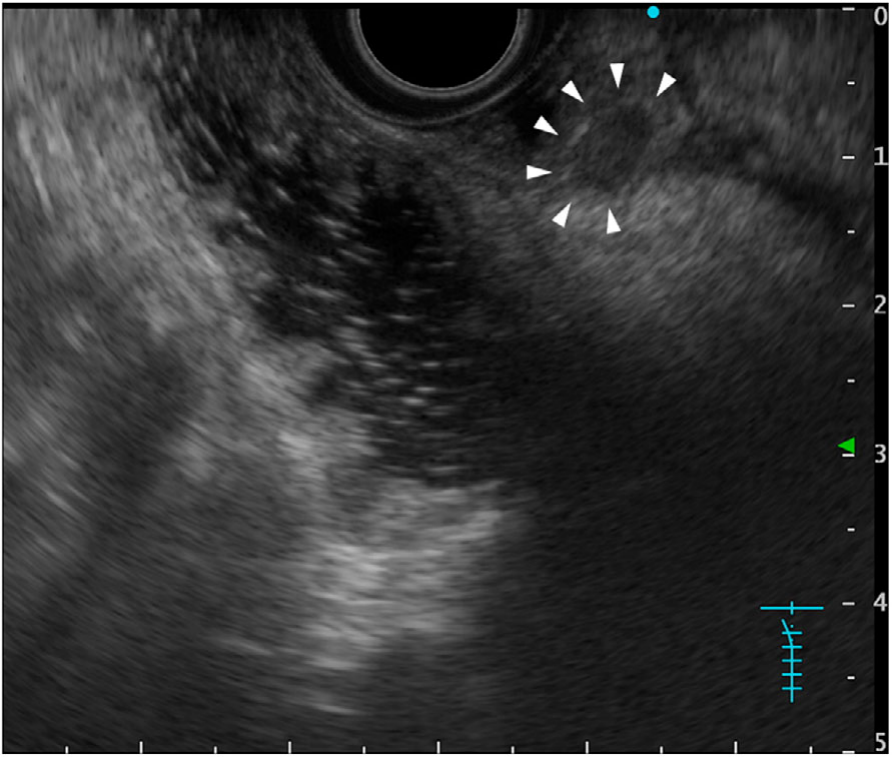
Endoscopic ultrasonography image. A 6.2‐mm hypoechoic tumor is seen within the submucosal layer of the ampulla of Vater (arrowhead). Endoscopic ultrasonography indicated no invasion to the muscularis propria layer of the duodenum and intraductal extension to the bile or pancreatic duct

Endoscopic ultrasonography showed a 6.2‐mm hypoechoic tumor within the submucosal layer of the ampulla of Vater (Figure 2) but no invasion of the muscularis propria layer of the duodenum or intraductal extension to the bile or pancreatic duct. In addition, no enlarged surrounding lymph nodes were observed, and enhanced multidetector computed tomography did not reveal any distant or lymph node metastases. Positron emission tomography did not reveal abnormal 18F‐fluorodeoxyglucose uptake.

**FIGURE 3:**
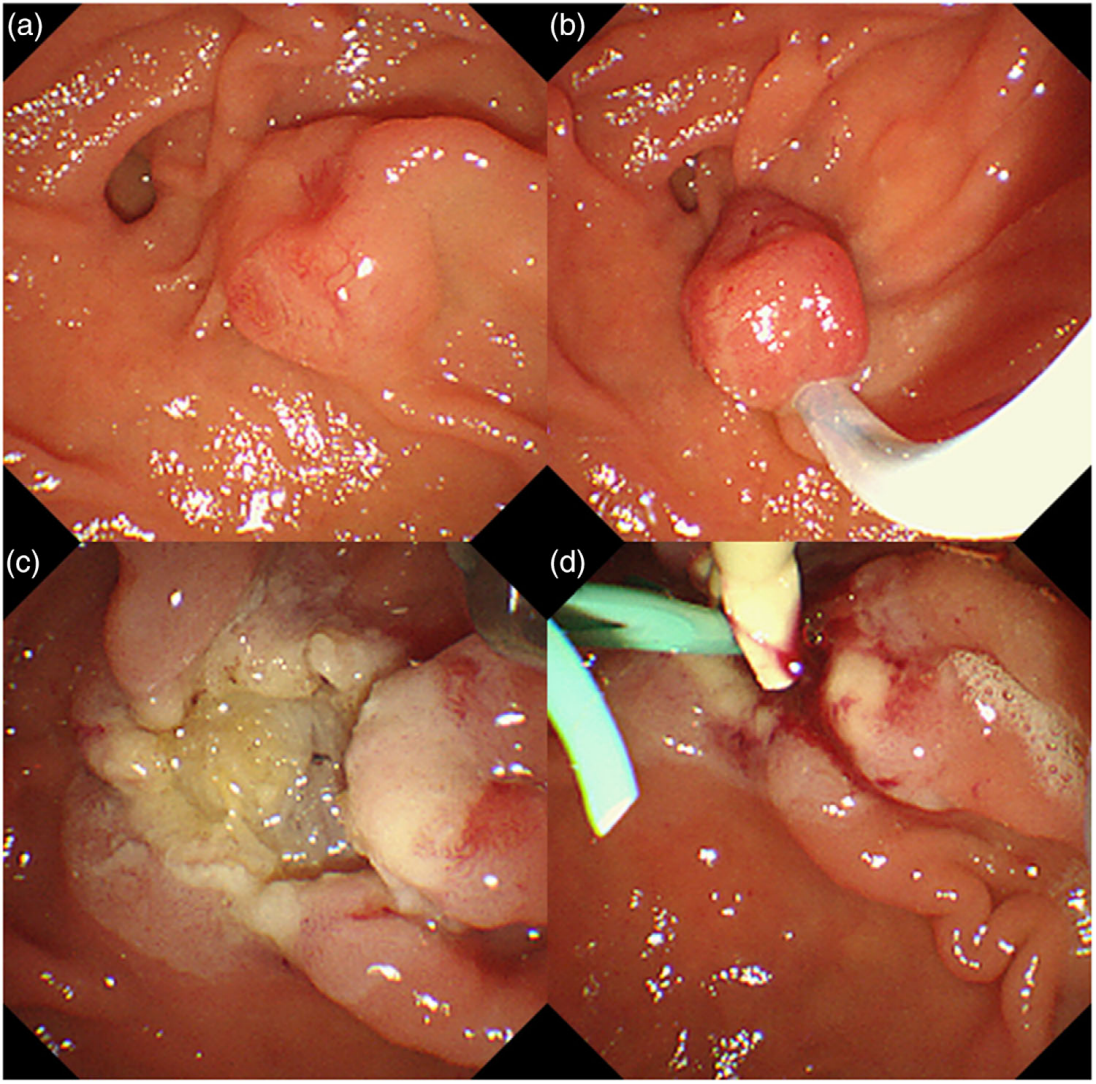
Endoscopic papillectomy was performed using a duodenoscope for ampullary neuroendocrine tumor. (a) Duodenoscopic view of ampullary neuroendocrine tumor. (b) Endoscopic snare papillectomy. (c) Post‐papillectomy ulcer. (d) After papillectomy, a pancreatic stent and a biliary plastic stent were placed to prevent possible pancreatitis and cholangitis

Accordingly, we selected endoscopic papillectomy as the treatment for the NET. Endoscopic papillectomy was performed using a duodenoscope (TJF‐260V; Olympus Medical Corp., Tokyo, Japan). We placed a snare (CAPTIVATOR II 15 mm; Boston Scientific Corp., Marlborough, MA, USA) and resected the lesion. En‐bloc resection was then performed (Figure 3). No bleeding occurred, and after the endoscopic papillectomy, a pancreatic stent (Geenen 5Fr, 5 cm; Cook Medical Japan, Tokyo, Japan) and a biliary plastic stent (Harmo Ray 7Fr, 7 cm; Hanaco Medical, Saitama, Japan) were placed to prevent possible pancreatitis and cholangitis. The anal side of the resected surface was closed using an EZ clip (Olympus Medical Corp.) to prevent postoperative bleeding. The patient experienced no postoperative adverse events, and the plastic stents were removed within a week.

**FIGURE 4:**
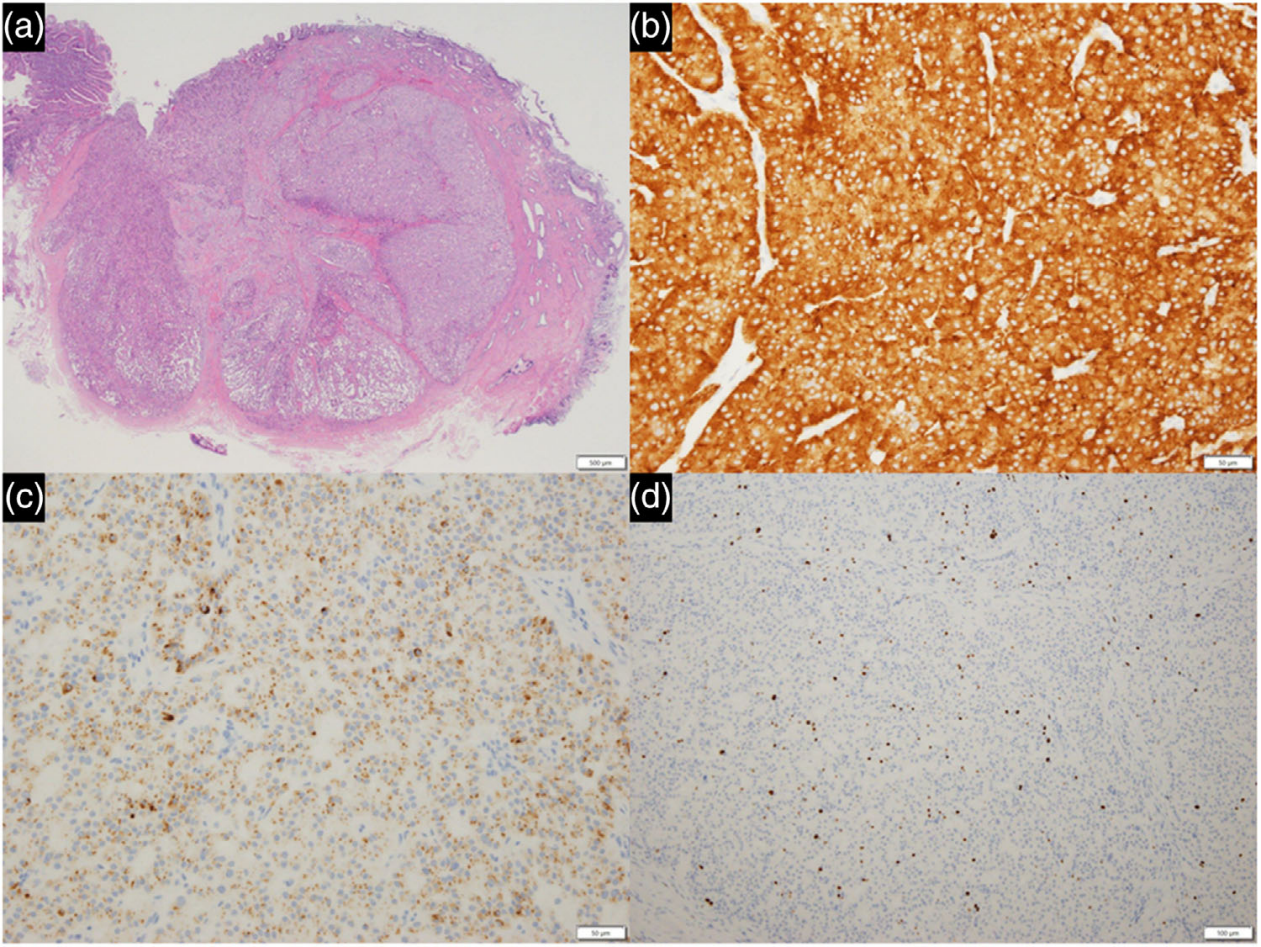
Histological and immunohistochemical findings of the resected specimen. (a) Hematoxylin‐eosin staining revealed that the tumor was 6.2‐mm in diameter with negative horizontal and vertical margins (Bar = 500 μm). (b) Positive synaptophysin staining (Bar = 50 μm). (c) Positive chromogranin A staining (Bar = 50 μm). (d) The Ki‐67 index was <3% (Bar = 100 μm)

The histological diagnosis of the resected specimen was a 6.2‐mm G1 NET located within the submucosal layer (Figure 4). Immunohistochemical staining showed that the lesion was positive for chromogranin and CD56. The MIB‐1 index positivity rate was <3%. There was no lymphovascular invasion, and the tumor's horizontal and vertical margins were negative. Therefore, the final pathological diagnosis was a NET G1.

The patient was discharged without any postprocedural adverse events, and no additional surgical treatment was required. No recurrence was observed during the 26 months of follow‐up.

## DISCUSSION

NET of the duodenum and ampulla of Vater are rare. Recent advances in endoscopy have increased the frequency of duodenal and ampullary NETs.^1^, ^2^ Ampullary NETs are often diagnosed at advanced stages and have a worse prognosis than duodenal NETs. Jaundice, abdominal pain, pancreatitis, and weight loss have been reported in many cases. In contrast, locally resected ampullary NET is reported to have a prognosis comparable to that of locally resected duodenal NETs.^3^ Some reports on endoscopic papillectomy for small ampullary NETs have been published. However, evidence is insufficient, and it is controversial whether endoscopic papillectomy for small ampullary NETs is appropriate.^6^ Long‐term prognosis is also unknown.

According to the European Neuroendocrine Tumor Society consensus guidelines, surgical treatment is offered for ampullary and periampullary NETs.^7^ Lymph node involvement is common for duodenal and periampullary carcinoid tumors, particularly those >1 cm in size. Therefore, resection with lymphadenectomy for these larger tumors is recommended.^8^ Post‐pancreatoduodenectomy mortality has decreased to <5% in recent years. However, the rate of postoperative complications, including leaks, sepsis, bleeding, and cardiac events remain at 50%.^9^ Surgical treatment places a heavy burden on patients, and there is an urgent need to verify the appropriateness of endoscopic therapy.

For patients with significant comorbidities and a tumor size <2 cm, endoscopic resection or surgical ampullectomy is recommended and should be performed by a highly experienced surgeon because of the high mortality rate.^3^, ^10^

In particular, ampullary NETs <1 cm in size have been reported to have a low likelihood of lymph node metastasis. Increasingly, there are reports that endoscopic papillectomy may be indicated if NETs are small (<10 mm), low‐grade (G1), do not have muscle layer and bile duct invasion, and have detailed investigation using CT, endoscopic ultrasonography, and other modalities before treatment.^5^, ^6^ After treatment, lymphatic and venous invasion are important pathological findings that determine whether the disease will recur.

No cases of recurrence after endoscopic papillectomy for ampullary NET have been reported thus far, and this treatment may be a viable option in selected cases. Although there are some published reports of endoscopic papillectomy for NETs >10 mm,^2^, ^10^ their follow‐up periods are insufficient. Therefore, while the evidence for papillectomy for NETs in the Vater region is inadequate, only lesions smaller than 10 mm should be targeted. Suppose surgery is not feasible owing to comorbidities or other reasons, and papillectomy is performed for large low‐grade NET. In such cases, it is crucial to perform a detailed examination before treatment and a pathological examination after treatment to evaluate lymphatic and vascular invasion.

The rates of adverse events associated with papillectomy are as follows: bleeding, 2%–30%; pancreatitis, 4%–20%; and perforation, 4%. Pancreatitis can be prevented by placing a pancreatic duct stent after an endoscopic papillectomy.^9^ Endoscopic papillectomy is a complex treatment modality that should be performed by or under the supervision of a skilled endoscopist.

In this case, detailed pretreatment observation revealed that the NET was small (<10 mm) and low‐grade (G1), with no muscle layer or bile duct invasion; therefore, endoscopic papillectomy was performed, and there were no complications. There was no recurrence at 26 months of follow‐up. A few case reports of lymph node metastasis, even in NETs as small as 10 mm,^5^ require detailed pathological examination, including the evaluation for lymphovascular invasion and more prolonged and rigorous follow‐up. The follow‐up interval and period after endoscopic treatment for ampullary NET are not precise. However, at our institute, endoscopy and CT were performed 6 months after treatment, followed by CT and endoscopy once a year. Moreover, there should be a long‐term follow‐up since NETs grow slowly, and there are reports of lymph node metastases from tumors <10 mm.

## CONFLICT OF INTEREST

None.
